# Cardiac Arrest due to Butane Gas Inhalation in an 18 Years Old Boy

**DOI:** 10.1155/2019/2461346

**Published:** 2019-08-22

**Authors:** Abdulaziz A. AlRabiah, Abdulmalik M. AlShamrani, Afnan A. AlMass

**Affiliations:** ^1^Department of Emergency Medicine, King Saud University, Riyadh, Saudi Arabia; ^2^Almaarefa University, Riyadh, Saudi Arabia; ^3^Emergency Medicine Consaltant, King Saud University Medical City, Riyadh, Saudi Arabia

## Abstract

An 18-year-old male smoker inhaled butane gas out of a pocket lighter with his friend for the purpose of changing his voice. He suddenly collapsed and lost his consciousness. Upon arrival to the Emergency Department, he was found pulseless with a rhythm of ventricular fibrillation. Cardiopulmonary resuscitation (CPR) was initiated according to the advanced cardiac life support (ACLS) protocol for three cycles until return of spontaneous circulation archived. After extubation, the patient was ataxic and had significant memory loss and severe confusion. Days later he improved and was discharged with walking aid for his ataxia and a plan to followup with the neurology team for magnetic resonance imaging (MRI) of the brain and electroencephalogram (EEG) as an outpatient.

## 1. Introduction

Butane gas is a part of the hydrocarbon group, which has the ability to cross any body tissue due to the high lipophilic characteristics [1]. Butane gas was reported as an inhaled substance of abuse for the purpose of voice change or/and sometimes euphoria [2–4]. It is available in pocket lighters, deodorants, or in-home gases [2]. It is a tragic cause of death in many cases usually due to refractory cardiac tachyarrhythmia by sensitizing the heart to catecholamines [3–5] and can cross to different organ tissues causing symptoms of toxicity [1, 5, 6].

Majority of the patients will die due to unwitnessed arrest [7] and delaying the appropriate resuscitation which has shown bad prognosis [6]. Early recognition and supportive treatment can make better outcome [5, 8].

## 2. Case Report

Our patient is an 18-year-old male smoker who is not known to have any medical illnesses. One day he was with his friend at home inhaling butane gas out of a pocket lighter for the purpose of enjoying voice change, and then suddenly he collapsed and became unresponsive at home, which was witnessed by his family, and he was taken immediately to the hospital, 10 minutes away from his house. Upon arrival at the emergency department (ED), he was found to be pulseless. Cardiopulmonary resuscitation (CPR) was initiated and continued for three cycles following standard advanced cardiovascular life support (ACLS) guidelines. Initially, the cardiac monitor showed a rhythm of ventricular fibrillation and he received two doses of 200 joules of unsynchronized shocks.

Return of spontaneous circulation (ROSC) was achieved and he was intubated using rapid sequence intubation (RSI). Chest X-ray, brain computed tomography (CT), and echocardiography were unremarkable to any abnormal findings. Blood investigations including complete blood count (CBC), metabolic panel, electrolytes, renal panel, and cardiac enzymes were all in the normal limits except for a mild metabolic acidosis in the venous blood gas.

Six hours from ROSC the patient was extubated in the intensive care unit (ICU). As he regained his consciousness after extubation he was found to be ataxic, disoriented, and confused. At that time the patient was referred to our facility as a tertiary care health center for higher care.

Upon presentation, to our hospital, the patient was vitally stable, confused, having short-term memory loss, ataxia, and disoriented to time and place.

**Figure 1:**
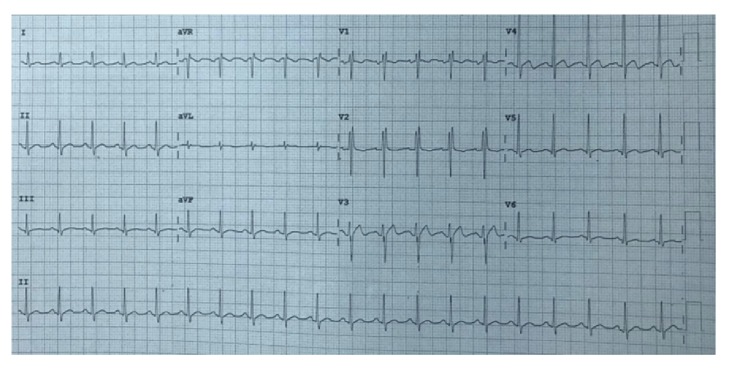
Patient ECG upon presentation to the hospital.

In the Emergency Department, multiple electrocardiographs (ECGs) were taken which showed multiple ST segment abnormalities (Figure 1). CT brain was repeated and showed no acute brain insult, and blood investigations were overall unremarkable.

The patient was assessed by the cardiologist and neurologist in the Emergency Department and the decision was made to admit him under cardiology care in the cardiac care unit (CCU) for rhythm monitoring and further workup. A multidisciplinary team was involved to look for the cause of the patient's neurological deficit.

The next day the neurology team performed a detailed neurological examination which showed the above-witnessed disorientation for time and place, short-term memory loss, and ataxia. At that time magnetic resonance imaging (MRI) and electroencephalogram (EEG) were ordered to rule out hypoxic brain injury vs. toxic brain injury.

By the fourth day in his hospital course, the patient improved, was conscious, oriented, and regained memory. He started mobilizing out of the bed with walking aid. No cardiac or neurological events were recorded during his hospital stay so he was discharged home with followup appointments for magnetic resonance imaging (MRI) and electroencephalogram (EEG) brain with neurology clinic.

## 3. Discussion

We present this case of butane inhalation out of pocket lighter gas for a young boy. Butane gas is volatile and quickly caused hypoxia, which is linked to causing cardiac tachyarrhythmias. It is also highly lipophilic which eases its crossing of the brain and cardiac tissue causing neurological deficit or cardiac arrhythmias. Published literature shows that these patients die of ventricular fibrillation or live with bad neurological outcomes due to the delay in resuscitation or unrecognition of the cause. Our patient was lucky to survive from this tragic event which may be due to the rapid initiation of resuscitation procedure.

## 4. Conclusion

Butane gas inhalation is linked with serious tragic outcomes ranging from transient cardiac arrhythmias to complete cardiac arrests and also involves multiple neurological outcomes. The public population must be educated about the dangers of this gas and its bad outcomes and should be recommended to stay away from it.
